# Prognostic Value of Baseline ^18^F-FDG PET/CT Functional Parameters in Patients with Advanced Lung Adenocarcinoma Stratified by EGFR Mutation Status

**DOI:** 10.1371/journal.pone.0158307

**Published:** 2016-06-23

**Authors:** Dalong Wang, Minghui Zhang, Xuan Gao, Lijuan Yu

**Affiliations:** 1 Department of Radiology, The Second Affiliated Hospital of Harbin Medical University, Harbin, Heilongjiang Province, China; 2 Department of Medical Oncology, Harbin Medical University Cancer Hospital, Harbin, Heilongjiang Province, China; 3 Department of PET/CT, Harbin Medical University Cancer Hospital, Harbin, Heilongjiang Province, China; Peking University People Hospital, CHINA

## Abstract

The study objective was to retrospectively analyze the metabolic variables derived from 18 F-fluorodeoxyglucose (^18^F-FDG) positron emission tomography/computed tomography (PET/CT) as predictors of progression-free survival (PFS) and overall survival (OS) in advanced lung adenocarcinoma stratified by epidermal growth factor receptor (EGFR) mutation status. A total of 176 patients (91, EGFR mutation; 85, wild-type EGFR) who underwent ^18^F-FDG PET/CT before treatment were enrolled. The main ^18^F-FDG PET/CT-derived variables: primary tumor maximum standardized uptake value (SUV_maxT_), primary tumor total lesion glycolysis (TLG_T_), the maximum SUV_max_ of all selected lesions in whole body determined using the Response Evaluation Criteria In Solid Tumors (RECIST) 1.1 criteria (SUV_maxWBR_), and whole-body total TLG determined using the RECIST 1.1 criteria (TLG_WBR_) were measured. Survival analysis regarding TLG_WBR,_ and other factors in advanced lung adenocarcinoma patients stratified using EGFR mutation status, were evaluated. The results indicated that high TLG_WBR_ (≥259.85), EGFR wild-type, and high serum LDH were independent predictors of worse PFS and OS in all patients with advanced lung adenocarcinoma. Among patients with wild-type EGFR, only TLG_WBR_ retained significance as an independent predictor of both PFS and OS. Among patients with the EGFR mutation, high serum LDH level was an independent predictor of worse PFS and OS, and high TLG_WBR_ (≥259.85) was an independent predictor of worse PFS but not worse OS. In conclusion, TLG_WBR_ is a promising parameter for prognostic stratification of patients with advanced lung adenocarcinoma and EGFR status; however, it cannot be used to further stratify the risk of worse OS for patients with the EGFR mutation. Further prospective studies are needed to validate our findings.

## Introduction

Lung cancer is the most common cancer and the leading cause of malignant diseases worldwide. Non-small-cell lung cancer (NSCLC) accounts for 80–85% of lung cancer cases [1]. Adenocarcinoma is the most frequently diagnosed histological subtype of primary lung cancer in most countries, accounting for almost half of all lung cancers. Most patients with lung adenocarcinomas are diagnosed with advanced disease, which is clinically aggressive and has high metastatic potential [2]. Despite remarkable advances in surgical resections and targeted therapies, the prognosis of lung adenocarcinoma patients remains poor [3]. Thus, identifying novel prognostic methods is very important in improving the predictive ability of outcomes for patients with lung adenocarcinoma.

Because of its advantages of noninvasive evaluation and accuracy, positron emission tomography and computed tomography (PET-CT), as assessed using the maximum standardized uptake value (SUV_max_), has been increasingly used for the assessment of the initial staging of NSCLC, restaging, recurrence, and monitoring the response to therapy [4–6]. The rationale for using FDG-PET in tumors is its ability to measure increased glucose metabolism in tumor cells. Recent studies have also shown that the degree of tumor FDG uptake (SUV_max_) regarding PET was a significant prognostic factor in NSCLC [7–9]. When analyzing the prognostic capability of FDG PET, the most commonly used method for the quantification of FDG uptake is SUV_max_ [10]. However, there are many disadvantages to the use of SUV_max_, particularly the variability introduced by the high statistical noise associated with single voxel analysis [11]. Total lesion glycolysis (TLG) not just denotes the SUV_max_ but also the tracer uptake of the entire lesion [12]. Despite several advantages of TLG over SUV_max_, further study is needed to verify if TLG is a better prognostic predictive factor.

The biological characteristics of patients with the epidermal growth factor receptor (EGFR) mutation differ greatly from those with wild-type EGFR; this leads to the use of different therapies and different clinical outcomes in patients with advanced (≥stage IIIB) lung adenocarcinoma. Consequently, in patients with advanced-stage disease, it is important to study the prognostic value of the EGFR mutation and wild-type EGFR, and to identify a new non-invasive, convenient, and practical predictor of outcome. To our knowledge, no previous studies have demonstrated the value of whole body TLG (lesions were selected in accordance with the Response Evaluation Criteria In Solid Tumors (RECIST) 1.1 criteria) in the prediction of progression-free survival (PFS) in advanced lung adenocarcinoma stratified using EGFR mutation status. Our study was designed to investigate the prognostic value of the ^18^F-FDG PET-derived parameter TLG per RECIST 1.1 criteria in patients with advanced lung adenocarcinoma stratified using EGFR mutation status.

## Materials and Methods

### Patient characteristics

All participants were examined at our PET/CT center at least 4 weeks before receiving any therapy. One hundred and seventy-six consecutive nonsurgical patients with histologically proven advanced stage (≥stage IIIB lung adenocarcinoma who underwent a ^18^F-FDG PET/CT scan before treatment) were included in this retrospective study (from February 2009 to October 2013). Patients with advanced lung adenocarcinoma were identified for inclusion in this study in accordance with the following criteria. (1) No brain metastasis (the characterization of brain metastasis was influenced by high physiological cerebral uptake of ^18^F-FDG). (2) No other types of concurrent cancer. (3) Known EGFR gene mutation status. (4) Unequivocal primary lung tumors with delineated borders. (5) Treatment according to the institutional guidelines, and clinical follow-up for at least 24 months in our hospital. The study was approved by the Ethics Committee of Harbin Medical University Cancer Hospital. All patients have provided oral consent forms for the use of their medical data. We could not obtain written informed consent from all participants as this was a retrospective study and the majority of the patients had been discharged from hospital at the time of analysis. The oral informed consent was documented in the electronic or paper patient file and approved by the local ethics committee. We collected and analyzed the data anonymously, and no results were ever connected to their identities.

Staging was performed according to the Union for International Cancer Control/the American Joint Committee on Cancer Staging System for NSCLC (UICC/AJCC) [13]. Tissue specimens obtained by conventional bronchoscope or CT-guided percutaneous transthoracic needle biopsy excision were used for EGFR gene detection. *EGFR* mutations (exons 18, 19, 20, and 21) and v-Ki-ras2 Kirsten rat sarcoma viral oncogene (*KRAS*) mutations (codons 12, 13, and 61) were analyzed using the amplification refractory mutation system [14]. For analysis of the *EML4* (echinodermmicrotubule-associated protein like 4)–*ALK* (anaplastic lymphoma kinase) gene fusion, we performed fluorescence in situ hybridization analysis for ALK immunohistochemistry-positive cases. According to the National Comprehensive Cancer Network (NCCN) guidelines [15], patients with mutant *EGFR* receive targeted drugs (gefitinib), while patients with rapidly progressive disease undergo second-line treatment including docetaxel or pemetrexed. For patients with wild-type *EGFR*, standard first-line treatment usually consists of platinum-based doublet chemotherapy, and second-line treatment options available to patients who experience failure of first-line treatment include additional chemotherapy (docetaxel and pemetrexed). Patients with *EML4*–*ALK* rearrangement receive targeted drugs (crizotinib), while patients with rapidly progressive disease undergo second-line treatment consisting of platinum-based doublet chemotherapy. The treatment of patients with mutant *KRAS* is the same as the treatment of patients with wild-type *EGFR*.

Data regarding clinicopathological characteristics, treatment, follow-up, and Eastern Cooperative Oncology Group (ECOG) performance status were recorded according to the patients’ medical records. The clinical data for each patient were discussed and determined by two oncologists. Lesions were selected in accordance with the Response Evaluation Criteria In Solid Tumors (RECIST) 1.1 criteria as previously described [16]. Briefly, pulmonary tumors with the longest diameter ≥10 mm, lymph nodes with the longest diameter ≥15 mm in the short axis, and metastatic solid lesions with the longest diameter ≥10 mm were selected. All measurable lesions up to a maximum of five in a single patient, and two lesions in one organ were recorded in our study.

### PET/CT acquisition

All patients fasted for 4–6 h. Blood glucose levels were checked in the peripheral blood (<150 mg/dL was considered normal) before the PET/CT examination. PET/CT images were obtained using an integrated PET/CT scanner (Discovery ST: GE Medical systems, Milwaukee, WI, USA) at 60 min after intravenous administration of ^18^F-FDG (5.55–7.40 MBq/kg). The scan range started at the mid-thighs and proceeded to the head. A whole-body unenhanced CT scan was performed using the following parameters: 140 kV, 150 mA, 0.8 s per rotation, 22.5 mm/s table speed, and slice thickness of 3.75 mm. Data from the CT scans were reconstructed from a 512 × 512 matrix to a 128 × 128 matrix to satisfactorily match the PET data and allow image fusion. The PET scan was carried out in the same position for all patients and using the two-dimensional imaging mode. PET image datasets were reconstructed using an iterative algorithm (the ordered subsets expectation maximization). The emission scan was obtained at 3 min per bed position, and six to seven bed positions were generally performed for all patients. All of the transaxial, sagittal, and coronal images were displayed and analyzed on a workstation (Xeleris; General Electric Medical Systems,Milwaukee, WI, USA).

All CT, PET, and PET/CT reconstructed images were loaded onto a workstation (Advanced workstation 4.6; GE Medical Systems, Milwaukee, WI, USA). After identification of the tumors, parameters were measured from the attenuation-corrected torso ^18^F-FDG PET/CT images and calculated semiautomatically using PET VCAR; the PET-based lesion contour was defined using a threshold of 40% of the tumoral SUV_max_, and the corresponding parameters were provided. If the defined tumor margin was not appropriate, relative to the fused CT, adjustment of the SUV_max_ threshold was required until a satisfactory tumoral outline was achieved. The volume boundaries were automatically drawn to incorporate each target lesion in the axial, coronal, and sagittal PET-CT images. In our study, the output results included the SUV_max_, metabolic tumor volume (MTV), and TLG of the tumor. ^18^F-FDG PET/CT images were assessed by two experienced nuclear medicine physicians with PET/CT imaging experience as well as familiarity with PET-VCAR software and our PACS system. These images were reviewed to localize the target lesions that had been confirmed by two nuclear medicine physicians; any discrepancies were resolved by consensus. For each patient, SUV_maxT_ was the SUV_max_ of the primary tumor. SUV_maxWBR_ was the maximum SUV_max_ of all selected lesions in the whole body determined using the RECIST 1.1 criteria. TLG was the MTV multiplied by the mean SUV of the tumor. TLG of the primary tumor (TLG_T_) was the MTV multiplied by the mean SUV of the primary lung tumor. TLG_WBR_ was the whole body TLG calculated as the sum of all corresponding TLG values of the lesions selected using the RECIST 1.1 criteria.

### Statistical analysis

The results of ^18^F-PET/CT were displayed as continuous variables. To test the consistency of the measurement of TLG_WBR_ on PET images, TLG_WBR_ were calculated independently twice by two groups of nuclear medicine physicians (any discrepancies were resolved by consensus in each group of two persons) per RECIST 1.1 in all patients. Reliability of TLG_WBR_ was measured using the intraclass correlation coefficient (ICC) generated by a two-way random-effects model with an absolute agreement definition and is reported as a point estimate with a 95% confidence interval (CI). ICC is interpreted as follows: an ICC of 0.00–0.20 indicates slight reproducibility; an ICC of 0.21–0.40, fair reproducibility; an ICC of 0.41–0.60, moderate reproducibility; an ICC of 0.61–0.80, substantial reproducibility; and an ICC of > 0.80, almost perfect reproducibility [17]. The cutoff values for the categorization of low and high TLG_WBR_, TLG_T_, SUV_maxT_, SUV_maxWBR_, and age were proceeded by R (version 3.3.2) with the package of survival ROC (version:1.0.3) (R Development Core Team, Vienna, Austria, http://www.R-project.org). The cutoff values for the categorization of low and high TLG_WBR_, TLG_T_, SUV_maxT_, SUV_maxWBR_, and age were determined using receiver operation characteristic (ROC) curve analyses. The optimal cut-off value was determined using the value representing the maximal area under the curve, and maximal sum of sensitivity and specificity; the same cut-off value for each parameter was applied to compare the PFS and overall survival (OS) in all group analyses. Univariate analysis of prognostic factors for PFS and OS was achieved using the Kaplan–Meier method, and the log-rank test was used to evaluate the significance of the differences between the survival curves, the Cox proportional hazards model that included significant univariate variables was used to determine independent prognostic factors for PFS and OS in multivariate survival analyses. Risk of death was estimated on the basis of hazard ratios and the 95% confidence interval was recorded. Sex, age, performance status, serum LDH level, serum CEA level, EGFR gene status, and ^18^F-FDG PET/CT-derived parameters were used for univariate and multivariate prediction of OS and PFS. Variables with a *P* value < 0.05 on univariate analysis were selected for multivariate analysis. To evaluate multi-collinearity between PET/CT parameters and the relationship between serum LDH and metabolic parameters, Spearman’s rank correlation coefficient was calculated. The SPSS 19.0 (SPSS Inc., Chicago, IL, USA) software package was used for the analysis. A two-tailed *P* value < 0.05 was considered to indicate statistical significance.

## Results

### Patient characters and EGFR genetic status

The 176 patients with advanced lung adenocarcinoma consisted of 72 men and 104 women with a median age of 61 (range, 30–87) years. A total of 91 (52%) patients with the EGFR mutation were found. Eleven *EGFR* mutation-negative patients with *KRAS* mutations and two *EGFR* mutation-negative patients with *EML4*–*ALK* rearrangement were identified. No co-occurrences of driver mutations were found. Patients with wild-type EGFR consisted of 38 men and 47 women with a median age of 61 (range, 30–84) years. Patients with the EGFR mutation consisted of 34 men and 57 women with a median age of 60 (range, 34–87) years. Their characteristics are summarized in Table 1.

**Table 1:** General characteristics of patients with advanced lung adenocarcinoma.

Characteristic	n(%)
**Age**	
**<64**	105(60)
**≥64**	71(40)
**Sex**	
**Male**	72(41)
**Female**	104(59)
**History of smoking**	
**+**	103(59)
**-**	73(41)
**Mutation(EGRF)**	
**+**	91(52)
**-**	85(48)
**ECOG performance status**	
**0/1**	122(69)
**2/3/4**	54(31)
**Serum CEA level**	
**High**	85(48)
**Normal**	91(52)
**Serum LDH level**	
**High**	28(16)
**Normal**	148(84)
**SUV**_**maxT**_	
**<11.86**	99(56)
**≥11.86**	77(44)
**SUV**_**maxWBR**_	
**<16.14**	99(56)
**≥16.14**	77(44)
**TLG**_**T**_	
**<104.65**	108(61)
**≥104.65**	68(39)
**TLG**_**WBR**_	
**<259.85**	99(56)
**≥259.85**	77(44)

Abbreviations: EGFR: epidermal growth factor receptor; ECOG: Eastern Cooperative Oncology Group; SUV_maxT_: primary tumor maximum standardized uptake value (SUV_max_); SUV_maxWBR_: the maximum SUV_max_ of all selected lesions in whole body determined using the Response Evaluation Criteria In Solid Tumors (RECIST) 1.1 criteria; TLG_T_: primary tumor total lesion glycolysis (TLG); TLG_WBR_: whole-body total TLG determined using the RECIST 1.1 criteria.

### ^18^F-FDG PET/CT-derived parameters in advanced lung adenocarcinoma patients

In 176 patients with advanced lung adenocarcinoma, the median TLG_WBR_ was 208.9 (range, 4.09–5038.04), the median TLG_T_ was 62.07 (range, 2.59–4578.27), the median SUV_maxWBR_ was 13.38 (range, 3.25–50.07), and the median SUV_maxT_ was 10.58 (range, 1.96–40.44). In 85 patients with wild-type EGFR, the median TLG_WBR_ was 305.59 (range, 4.09–5038.04), the median TLG_T_ was 55.75 (range, 4.09–4578.27), the median SUV_maxWBR_ was 12.27 (range, 3.81–49.56), and the median SUV_maxT_ was 9.10 (range, 1.96–40.40). In 91 patients with the EGFR mutation, the median TLG_WBR_ was 182.11 (range, 5.29–2575.26), the median TLG_T_ was 67.30 (range, 2.59–1172.83), the median SUV_maxWBR_ was 13.47 (range, 3.25–50.07), and the median SUV_maxT_ was 12.14 (range, 2.03–40.44).

**Fig 1:**
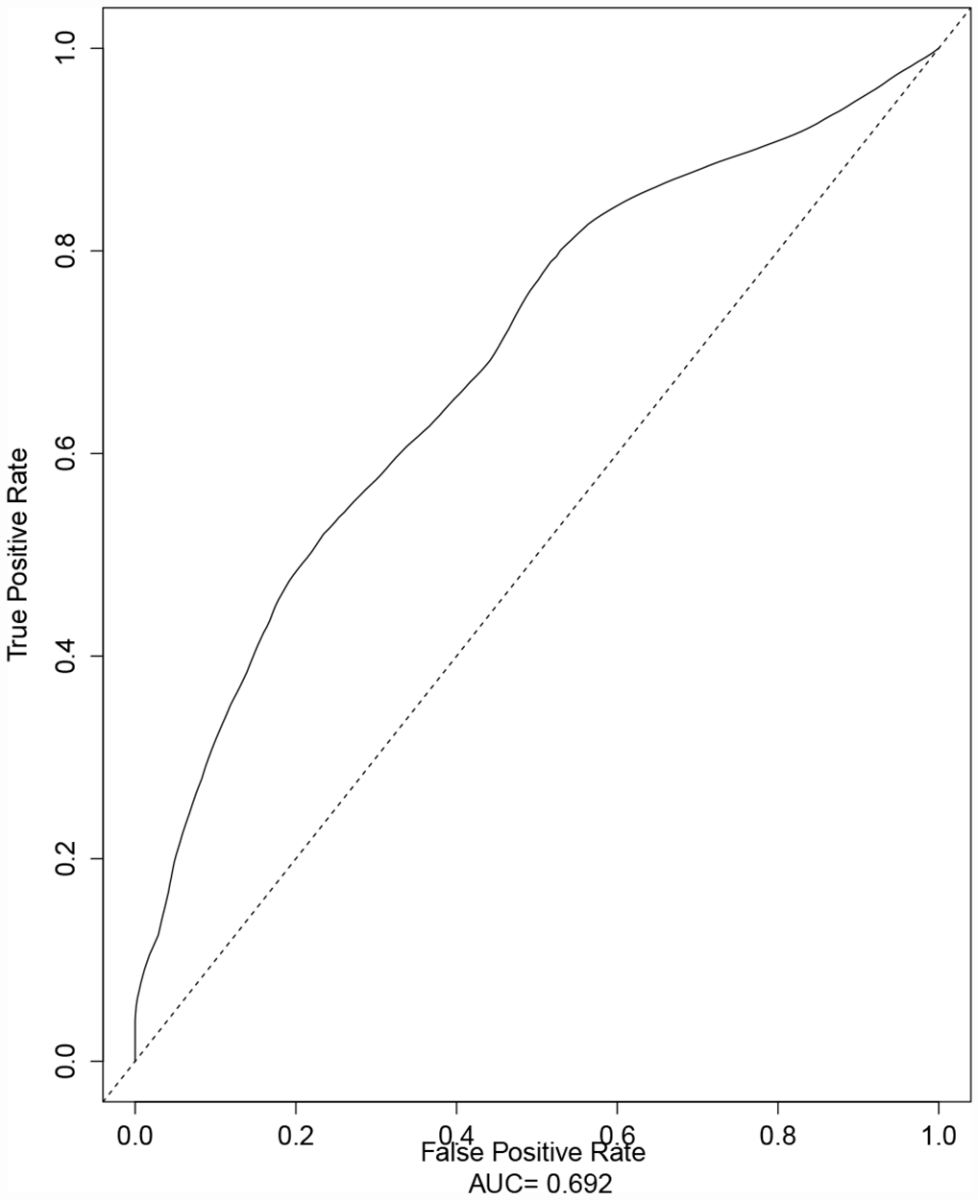
ROC curve for the identification of the optimal cutoff point for whole body total lesion glycolysis (TLG_WBR_) in advanced lung adenocarcinoma patients. The optimal cutoff value for TLG_WBR_ determined using the Response Evaluation Criteria In Solid Tumors 1.1 criteria was 258.98, and the area under the curve with the maximum summation of sensitivity and specificity was 0.69.

The ICC (the reliability between two group of nuclear medicine physicians with respect to TLG_WBR_ on PET per RECIST 1.1 in all patients) was 0.972 (95% CI: 0.963, 0.979). Because the TLG is calculated by multiplying the MTV and mean SUV, multi-collinearity between MTV and TLG was evaluated. The result of the Spearman’s rank correlation test showed a significant correlation between MTV_WBR_ and TLG_WBR_ (r = 0.898; *P*<0.0001). Spearman’s rank correlation analyses showed a correlation between serum LDH level and TLG_WBR_ (r = 0.205; *P* = 0.006). No such correlation was identified between serum LDH level and SUV_maxT,_ SUV_maxWBR_, or TLG_T_ (r = −0.037, *P* = 0.624; r = 0.079, *P* = 0.296; and r = −0.004, *P* = 0.958, respectively). In 91 patients with the *EGFR* mutation, the LDH level was also correlated with TLG_WBR_ (r = 0.210; *P* = 0.046); no such correlation was found between serum LDH level and TLG_WBR_ (r = 0.187; *P* = 0.086) in 85 patients with wild-type *EGFR*. According to the ROC analysis, the cutoff points for the categorization of low and high SUV_maxT_, SUV_maxWBR_, TLG_T_, TLG_WBR_, and age values were 11.68, 16.14, 104.65, 259.85, and 64, respectively. The ROC curve for identifying the optimal cutoff point of TLG_WBR_ in advanced lung adenocarcinoma patients is shown in Fig 1.

### Survival analysis in 176 patients with advanced lung adenocarcinoma

**Fig 2:**
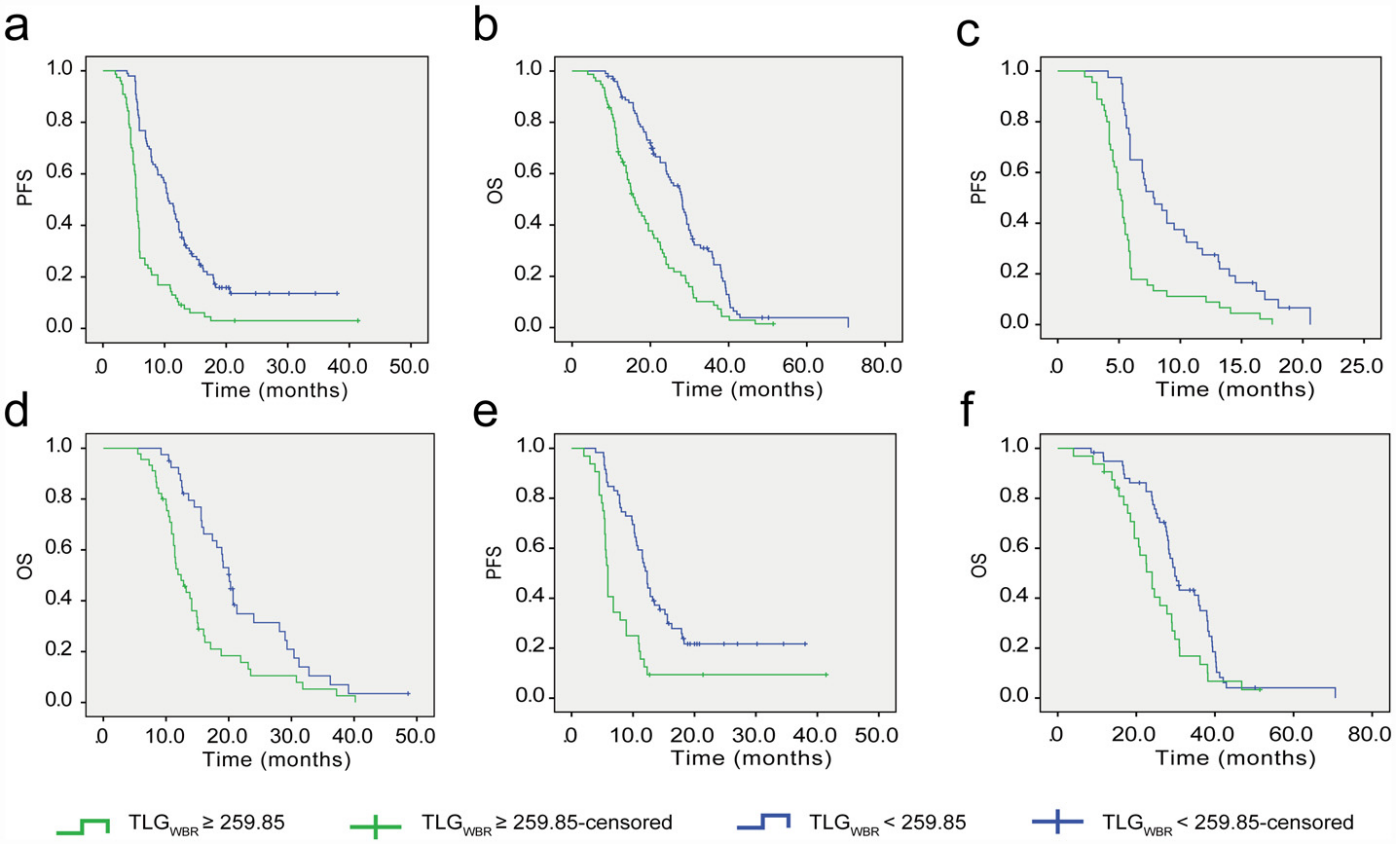
Kaplan–Meier survival analysis. **a** progression-free survival (PFS) and **b** overall survival (OS) according to whole-body total lesion glycolysis (TLG_WBR_) determined using the Response Evaluation Criteria In Solid Tumors 1.1 criteria in 176 patients with advanced lung adenocarcinoma. Kaplan–Meier analysis of **c** PFS and **d** OS according to TLG_WBR_ in 85 patients with wild-type EGFR. **e** Kaplan–Meier analysis of PFS and **f** OS according to TLG_WBR_ in 91 patients with the EGFR mutation.

**Fig 3:**
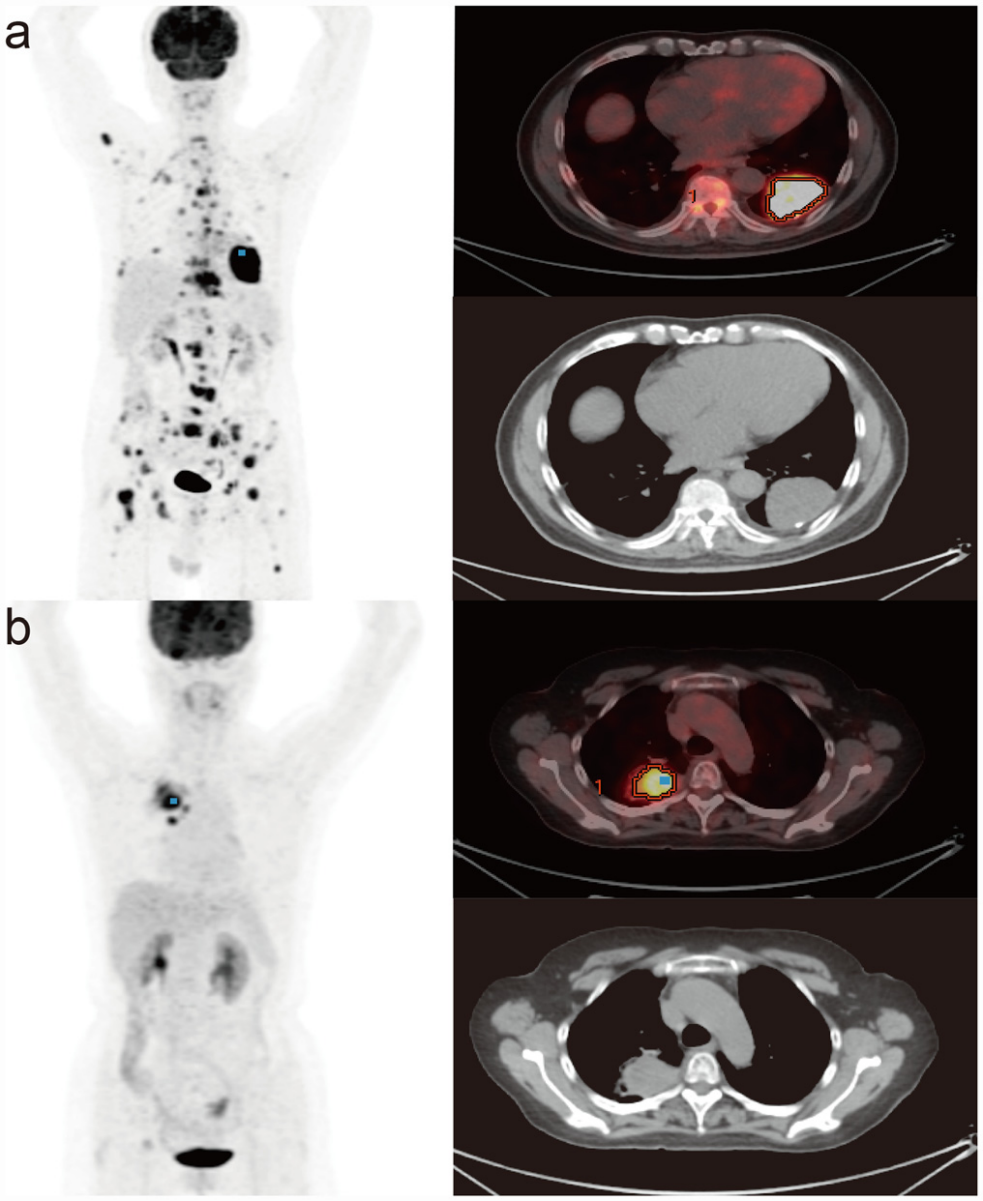
^18^F-FDG PET/CT in patients with advanced lung adenocarcinoma. **a** A 71-year-old man with high whole-body total lesion glycolysis (TLG_WBR_) determined using the Response Evaluation Criteria In Solid Tumors 1.1 criteria (7944.00) and primary tumor total lesion glycolysis (TLG_T_) (1250.46). Progression-free survival (PFS) was 5.9 months and overall survival (OS) was 11.9 months. EGFR mutation status was the wild-type. **b** A 68-year-old woman with low TLG_WBR_ (179.74) and TLG_T_ (142.41). PFS was 12.3 months and OS was 35.9 months. EGFR mutation status involved the mutation of exon 19 (delE746-A750).

The median PFS and OS for 176 patients in this study were7.4 (range, 2.0–41.4) months and 20.7 (range, 4.0–70.6) months, respectively. Among all patients, univariate survival analysis showed that high TLG_WBR_ (≥259.85), high TLG_T_ (≥104.65), high SUV_maxWBR_ (≥16.14), EGFR wild-type, high serum LDH level, and poor ECOG performance status were positively correlated with worse PFS. High TLG_WBR_ (≥259.85), high SUV_maxWBR_ (≥16.14), EGFR wild-type, high serum LDH level, poor ECOG performance status, and male sex were positively correlated with worse OS (Table 2). The Kaplan–Meier survival curves for PFS and OS in 176 patients with advanced lung adenocarcinoma according to TLG_WBR_ are presented in Fig 2a and 2b. Multivariate survival analysis showed that high TLG_WBR_ (≥259.85), presence of wild-type EGFR, and high serum LDH level were independent predictors of worse PFS and OS (Table 3). Representative examples from patients with advanced lung adenocarcinoma who had low and high TLG_WBR_ values are shown in Fig 3.

**Table 2:** Univariate analysis of progression-free survival and overall survival in 176 patients with advanced lung adenocarcinoma.

Parameter	No. of patients	Progression-free survival		Overall survival	
		Mean survival time(months ±SE)	*P* value	Mean survival time(months ±SE)	*P* value
**Age**					
**<64**	105	10.16±1.07	0.311	23.06±1.42	0.606
**≥64**	71	11.95±1.05		24.95±1.25	
**Sex**					
**Male**	72	10.11±1.08	0.192	21.30±1.39	0.044
**Female**	104	11.75±1.01		26.11±1.23	
**Smoker**					
**+**	103	9.49±0.71	0.057	22.52±1.14	0.128
**-**	73	12.87±1.33		26.48±1.55	
**ECOG performance status**					
**0/1**	122	12.52±1.02	0.003	26.18±1.18	0.006
**2/3/4**	54	7.83±0.65		19.84±1.58	
**Serum CEA level**					
**Normal(≤5)**	91	11.33±1.12	0.972	23.94±1.48	0.555
**High(>5)**	85	10.79±0.97		24.70±1.25	
**Serum LDH level**					
**High**	28	6.13±0.65	0.000	18.34±2.02	0.002
**Normal**	148	12.41±0.94		25.58±1.12	
**Mutation**					
**EGRF(+)**	91	14.64±1.36	0.000	29.59±1.36	0.000
**EGRF(-)**	85	7.76±0.49		18.38±1.06	
**SUV**_**maxT**_					
**<11.86**	99	10.49±0.88	0.817	24.58±1.42	0.998
**≥11.86**	77	12.01±1.34		23.96±1.30	
**SUV**_**maxWBR**_					
**<16.14**	99	12.48±1.00	0.002	26.79±1.44	0.007
**≥16.14**	77	9.61±1.16		21.32±1.24	
**TLG**_**T**_					
**<104.65**	108	12.05±0.99	0.026	26.21±1.34	0.055
**≥104.65**	68	9.79±1.15		21.38±1.35	
**TLG**_**WBR**_					
**<259.85**	99	13.99±1.10	0.000	28.37±1.35	0.000
**≥259.85**	77	7.39±0.79		19.11±1.20	

Abbreviations: ECOG: Eastern Cooperative Oncology Group; EGFR: epidermal growth factor receptor; SUV_maxT_: primary tumor maximum standardized uptake value (SUV_max_); SUV_maxWBR_: the maximum SUV_max_ of all selected lesions in whole body determined using the Response Evaluation Criteria In Solid Tumors (RECIST) 1.1 criteria; TLG_T_: primary tumor total lesion glycolysis (TLG); TLG_WBR_: whole-body total TLG determined using the RECIST 1.1 criteria.

**Table 3:** Multivariate analysis of progression-free survival and overall survival in 176 patients with advanced lung adenocarcinoma.

Parameter	Progression-free survival			Overall survival		
	Hazard ratio(exp. B)	95%CI	*P* value	Hazard ratio(exp. B)	95%CI	*P* value
**SUV**_**maxWBR**_**≥16.14**	1.466	1.043–2.061	0.280	1.381	0.976–1.952	0.068
**TLG**_**T**_**≥104.65**	1.001	0.706–1.419	0.995			
**TLG**_**WBR**_**≥259.85**	1.896	1.296–2.775	0.001	1.579	1.089–2.289	0.016
**EGFR(-)**	1.763	1.262–2.462	0.001	2.502	1.773–3.530	0.000
**Serum LDH (high)**	2.021	1.294–3.156	0.002	1.620	1.007–2.606	0.047
**ECOG performance status(0/1)**	1.381	0.965–1.975	0.077	1.185	0.814–1.727	0.375
**Sex (male)**				0.722	0.516–1.012	0.058

Abbreviations: SUV_maxWBR_: the maximum SUV_max_ of all selected lesions in whole body determined using the Response Evaluation Criteria In Solid Tumors (RECIST) 1.1 criteria; TLG_T_: primary tumor total lesion glycolysis (TLG); TLG_WBR_: whole-body total TLG determined using the RECIST 1.1 criteria; EGFR: epidermal growth factor receptor; ECOG: Eastern Cooperative Oncology Group.

### Prognostic value of TLG_WBR_ in 85 patients with wild-type EGFR

Univariate survival analysis indicated a significant association between high TLG_WBR_ (≥259.85), high TLG_T_ (≥104.65), high serum LDH, male sex, and worse PFS and OS (Table 4). The Kaplan–Meier survival curves for PFS and OS of patients with wild-type EGFR according to TLG_WBR_ are shown in Fig 2c and 2d. However, on multivariate analysis only TLG_WBR_ remained significant for both PFS and OS (Table 5).

**Table 4:** Univariate analysis of progression-free survival and overall survival in patients with wild-type EGFR.

Parameter	No. of patients	Progression-free survival		Overall survival	
		Mean survival time(months ±SE)	*P* value	Mean survival time(months ±SE	*P* value
**Age**					
**<64**	48	7.58±0.73	0.973	18.16±1.34	0.751
**≥64**	37	7.83±0.63		18.72±1.76	
**Sex**					
**Male**	38	6.65±0.67	0.038	15.46±1.44	0.009
**Female**	47	8.62±0.65		20.60±1.42	
**Smoker**					
**+**	55	7.54±0.64	0.606	19.30±1.61	0.543
**-**	30	8.21±0.76		17.92±1.39	
**ECOG performance status**					
**0/1**	49	8.40±0.69	0.147	19.20±1.37	0.497
**2/3/4**	36	6.94±0.66		17.32±1.72	
**Serum CEA level**					
**Normal(≤5)**	60	7.74±0.51	0.948	18.17±1.18	0.711
**High(>5)**	25	7.69±1.08		18.90±2.31	
**Serum LDH level**					
**High**	14	5.54±0.95	0.007	18.34±2.02	0.048
**Normal**	71	8.20±0.54		13.78±2.41	
**TLG**_**T**_					
**<104.65**	51	8.55±0.70	0.026	20.27±1.54	0.024
**≥104.65**	34	6.61±0.59		15.73±1.35	
**TLG**_**WBR**_					
**<259.85**	40	9.74±0.77	0.000	22.09±1.57	0.001
**≥259.85**	45	6.02±0.51		15.71±1.26	

Abbreviations: ECOG: Eastern Cooperative Oncology Group; TLG_T_: primary tumor total lesion glycolysis (TLG); TLG_WBR_: whole-body total TLG determined using the Response Evaluation Criteria In Solid Tumors 1.1 criteria.

**Table 5:** Multivariate analysis of progression-free survival and overall survival in patients with wild-type EGFR.

Parameter	Progression-free survival			Overall survival		
	Hazard ratio(exp. B)	95%CI	*P* value	Hazard ratio(exp. B)	95%CI	*P* value
**TLG**_**T**_**≥104.65**	1.229	0.766–1.973	0.393	1.381	0.847–2.252	0.195
**TLG**_**WBR**_**≥259.85**	2.128	1.290–3.509	0.003	1.881	1.127–3.139	0.016
**Serum LDH (high)**	1.432	0.772–2.656	0.255	1.181	0.589–2.369	0.640
**Sex (male)**	0.701	0.444–1.105	0.126	0.569	0.355–0.912	0.019

Abbreviations: TLG_T_: primary tumor total lesion glycolysis (TLG); TLG_WBR_: whole-body total TLG determined using the Response Evaluation Criteria In Solid Tumors 1.1 criteria.

### Prognostic values of TLG_WBR_ in 91 patients with the EGFR mutation

Univariate survival analysis revealed that a worse PFS was positively correlated with high TLG_WBR_ (≥259.85), high serum LDH level, and high serum CEA level. Worse OS appeared to be positively correlated with high TLG_WBR_ (≥259.85) and high serum LDH (Table 6). The Kaplan–Meier survival curves for PFS and OS in patients with the EGFR mutation patients according to TLG_WBR_ are shown in Fig 2e and 2f. Multivariate survival analysis revealed that high serum LDH level was an independent predictor of worse PFS and OS, and high TLG_WBR_ (≥259.85) was an independent predictor of worse PFS but not worse OS (Table 7).

**Table 6:** Univariate analysis of progression-free survival and overall survival in patients with the EGFR mutation.

Parameter	No. of patients	Progression-free survival		Overall survival	
		Mean survival time(months ±SE)	*P* value	Mean survival time(months ±SE)	*P* value
**Age**					
**<64**	57	13.27±2.04	0.504	30.45±1.65	0.503
**≥64**	34	14.77±1.61		27.42±1.93	
**Sex**					
**Male**	34	13.90±1.90	0.869	27.68±1.85	0.494
**Female**	57	14.05±1.64		30.22±1.66	
**Smoker**					
**+**	48	11.57±1.19	0.147	27.79±1.52	0.344
**-**	43	16.34±2.09		30.95±2.02	
**ECOG performance status**					
**0/1**	73	14.98±1.51	0.215	30.57±1.49	0.236
**2/3/4**	18	9.58±1.35		25.20±3.03	
**Serum CEA level**					
**Normal(≤5)**	31	18.72±2.65	0.049	33.31±2.30	0.078
**High(>5)**	60	11.75±1.18		27.07±1.38	
**Serum LDH level**					
**High**	14	6.71±0.91	0.000	21.87±2.81	0.002
**Normal**	77	16.09±1.54		31.07±1.48	
**TLG**_**T**_					
**<104.65**	57	14.91±1.57	0.215	30.87±1.66	0.506
**≥104.65**	34	12.98±2.09		26.77±1.95	
**TLG**_**WBR**_					
**<259.85**	59	16.66±1.57	0.000	31.83±1.61	0.018
**≥259.85**	32	9.84±1.85		24.62±1.87	

Abbreviations: ECOG: Eastern Cooperative Oncology Group; TLG_T_: primary tumor total lesion glycolysis (TLG); TLG_WBR_: whole-body total TLG determined using the Response Evaluation Criteria In Solid Tumors 1.1 criteria.

**Table 7:** Multivariate analysis of progression-free survival and overall survival in patients with the EGFR mutation.

Parameter	Progression-free survival			Overall survival		
	Hazard ratio(exp. B)	95%CI	*P* value	Hazard ratio(exp. B)	95%CI	*P* value
**TLG**_**WBR**_**≥259.85**	2.197	1.305–3.696	0.003	1.532	0.949–2.472	0.086
**Serum CEA (high)**	1.355	0.806–2.278	0.251			
**Serum LDH (high)**	2.215	1.177–4.166	0.014	2.128	1.149–3.941	0.014

Abbreviations: TLG_WBR_: whole-body total lesion glycolysis determined using the Response Evaluation Criteria In Solid Tumors 1.1 criteria.

## Discussion

The variation in the survival of patients with advanced lung adenocarcinoma is associated with multiple factors (EGFR mutation status, ECOG performance status, metabolism variables, serum markers, and sex). ^18^F-FDG PET/CT is a promising method and provides parameters for the selection of patients who have a better prognosis. In contrast to conventional methods, this approach for selecting patients could reveal the metabolism-specific differences in the prognostic value of PET/CT.

To the best of our knowledge, the current study involves the largest number of cases of any clinical study date, regarding the analysis of the prognostic significance of metabolic and volumetric parameters derived from ^18^F-FDG PET/CT in advanced lung adenocarcinoma patients with the EGFR mutation. Our study showed that TLG_WBR_ (TLG_WBR_ ≥259.85), EGFR mutation status, and serum LDH level for baseline ^18^F-FDG PET/CT were significant independent prognostic factors for PFS and OS in 176 patients with advanced lung adenocarcinoma. Other studies have reported that whole-body TLG was an independent prognostic factor in advanced lung adenocarcinoma patients with the EGFR mutation [18–19]. It should be noted that we have used TLG_WBR_ to represent whole-body metabolic tumor burden by selecting tumors in accordance with the RECIST 1.1 criteria. In addition, we analyzed the LDH level, which has not been previously been considered as a prognostic factor for PFS and OS; in our study, high serum LDH level was found to be an independent predictor of worse OS and PFS for all advanced lung adenocarcinoma patients. Furthermore, we found that high LDH serum level was an independent predictor of PFS and OS in patients with the EGFR mutation; this may have been because serum LDH level is related to intratumoral angiogenesis, tumor invasion ability, and resistance to therapy [20–21]. A previous study has reported that LDH levels had a significant effect on PFS in patients treated with erlotinib [22]. The present study also indicated that LDH level exhibited a correlation with TLG_WBR_ in the patients with advanced lung adenocarcinoma. However, no such correlation was found between serum LDH level and other PET/CT parameters (SUV_maxT,_ SUV_maxWBR_, TLG_T_). Furthermore, this correlation was mainly shown in patients with the *EGFR* mutation.

Regarding the correlation between the PET/CT metabolic parameters (SUV_max_, primary TLG, and whole-body TLG) and survival in patients with advanced NSCLC, there has been some disagreement among researchers. Some studies have reported that whole-body TLG is a better prognostic predictor of worse OS and PFS in patients with advanced NSCLC [23–24], whereas other studies have found no such correlation [25].

SUV_max_ as a metabolic parameter derived from PET/CT is easily quantified and widely used; nevertheless, SUV_maxT_ and SUV_maxWBR_ only denote the highest metabolic activity within the tumor, and do not consider the tumor extent [26–27]. In our study, SUV_maxT_ and SUV_maxWBR_ did not show any significant prognostic value as an independent predictor. This finding is consistent with those of recent studies [19, 24, 28]. In contrast, previous studies have revealed that SUV_maxT_ or SUV_maxWBR_ were independent predictors of survival in NSCLC [12, 29]. In contrast to SUV_max_, TLG incorporates the metabolic burden and disease extent and may provide a higher predictive value. The results of our study revealed that whole-body tumor glycolysis represented by TLG_WBR_ was a strong predictor of survival, which was similar to previously reported findings [18, 23–24]. However, our study indicated that total glycolysis in the primary tumor alone (TLG_T_) was not an independent predictor of survival, in line with some recent studies [19, 30]. The cause of this may be related to the fact that the TLG_T_, although accounting for the metabolic burden of the primary tumor, does not take the metabolic burden of the metastatic tumors into account. This finding contradicts previous reports suggesting that FDG uptake (TLG_T_) in the primary tumor was an independent prognostic factor [24, 31].

To date, only one other study has investigated the predictive value of metabolic and volumetric variables concerning the use of pretreatment ^18^F-FDG PET/CT in advanced lung adenocarcinoma patients stratified by EGFR mutation status using the RECIST 1.1 criteria [30]. In our study, the result of almost perfect reproducibility between nuclear medicine physicians with respect to TLG_WBR_ on PET per RECIST 1.1 criteria was found. In patients with wild-type EGFR, we found that TLG_WBR_ is an independent predictor of OS, which is consistent with findings of another study involving fewer (56) patients, and only OS as an endpoint [30]. In our study, we evaluated both PFS and OS as endpoints, because in a previous study regarding advanced NSCLC, PFS and OS were not confirmed to be consistently correlated [32]. It was found that TLG_WBR_ was an independent predictor of PFS both in patients with wild-type EGFR and the EGFR mutations. In contrast, TLG_WBR_ had no statistical significance as an independent predictor of OS in patients with the EGFR mutation. These findings will need to be validated in a large-scale cohort prospective study.

We have used the TLG_WBR_ of selected tumors (selected using the RECIST 1.1 criteria) as a substitute for summing up the TLG values for whole-body tumors [30]. In contrast to previous studies, whole-body TLG for TLG_WBR_ was strictly selected using RECIST 1.1 criteria, and the ICC indicated almost perfect reproducibility, as measured by the two groups of nuclear medicine physicians with respect to TLG_WBR_, although not all tumoral lesions could be included. The uptake of ^18^F-FDG is falsely influenced by inflammatory lesions and other lesions with obscure boundaries on images, making it difficult to distinguish between tumors and other nontumoral tissues; using our approach, this problem was relatively diminished and thus the outcomes were relatively credible. TLG_WBR_ could be applied in some cases with fewer limitations in a clinical setting. Conversely, the clinical application of the PERCIST 1.0 criteria may be influenced by a high incidence of hepatitis in China; this is because its reference tissue value relies on FDG uptake by the liver [33]. In addition, according to NCCN guidelines [16], *KRAS* mutation testing is not conventionally recommended, and targeted therapy is not currently available for patients with mutant *KRAS*, thus most patients did not undergo *KRAS* status tests in our study. The prognostic influence of *KRAS* mutations could not be analyzed for either PFS or OS in this study. In a previous study, no (0.0% (0/945)) lung adenocarcinoma patients had *KRAS* mutations with *EGFR* mutations, while one patient (0.1% (1/897)) had *EML4*–*ALK* and *EGFR* mutations in Asian populations [34], thus the very low proportion (nearly zero) (co-occurrences of driver mutations) almost could not influence the prognosis of patients receiving TKIs in this study. Furthermore, NCCN guidelines indicate that *KRAS* mutation does not appear to affect chemotherapeutic efficacy. The number of positive cases was too small to enable the analysis of prognosis of patients with *EML4*–*ALK* rearrangement. Therefore, our results could not be potentially influenced by *KRAS* mutation and *EML4*–*ALK* rearrangement. Further study is needed to analyze the influence of *KRAS* mutations or *EML4*–*ALK* rearrangement on prognosis.

Our study had a number of limitations. First, it was a single-center retrospective study, which could have led to various biases. In most patients, ^18^F-FDG PET/CT scans were only performed once, and the treatment-related response changes with TLG_WBR_ values were not evaluated. Second, not all the lymph node or distant metastases histopathology was evaluated. Third, all of the malignant lesions could not be included within the measurement of the PET workstation; thus, it was possible to underestimate the whole tumor burden using the imaging method. However, we selected the largest tumor metabolism burden per RESIST 1.1 as possible to avoid large inter-observer variation. Fourth, because there is no uniform or optimal threshold to delineate an accurate tumor boundary [18], a commonly adopted threshold (40% of the maximum SUV_max_) was used to determine tumor volume; this has been used in most of the previous studies [19, 27]. These limitations notwithstanding, our current data revealed that the TLG_WBR_ for selected tumors (using the RESIST 1.1 criteria) may be a promising parameter in the evaluation of the prognosis for advanced lung adenocarcinoma patients.

## Conclusion

TLG_WBR_ provides a strong prognostic indicator and could be an important guide for making treatment decisions in advanced lung adenocarcinoma patients with EGFR mutation status; however, it cannot be used to further stratify the risk of worse OS for patients with the EGFR mutation.
